# BAC-based cellular model for screening regulators of BDNF gene transcription

**DOI:** 10.1186/1471-2202-15-75

**Published:** 2014-06-18

**Authors:** Kaur Jaanson, Mari Sepp, Tamara Aid-Pavlidis, Tõnis Timmusk

**Affiliations:** 1 Department of Gene Technology, Tallinn University of Technology, Akadeemia tee 15, 12618 Tallinn, Estonia

**Keywords:** BDNF, Cell line, Bacterial artificial chromosome, HDAC inhibitor

## Abstract

**Background:**

Brain derived neurotrophic factor (BDNF) belongs to a family of structurally related proteins called neurotrophins that have been shown to regulate survival and growth of neurons in the developing central and peripheral nervous system and also to take part in synaptic plasticity related processes in adulthood. Since BDNF is associated with several nervous system disorders it would be beneficial to have cellular reporter system for studying its expression regulation.

**Methods:**

Using modified bacterial artificial chromosome (BAC), we generated several transgenic cell lines expressing humanised *Renilla* luciferase (hRluc)-EGFP fusion reporter gene under the control of rat *BDNF* gene regulatory sequences (rBDNF-hRluc-EGFP) in HeLa background. To see if the hRluc-EGFP reporter was regulated in response to known regulators of BDNF expression we treated cell lines with substances known to regulate *BDNF* and also overexpressed transcription factors known to regulate *BDNF* gene in established cell lines.

**Results:**

rBDNF-hRluc-EGFP cell lines had high transgene copy numbers when assayed with qPCR and FISH analysis showed that transgene was maintained episomally in all cell lines. Luciferase activity in transgenic cell lines was induced in response to ionomycin-mediated rise of intracellular calcium levels, treatment with HDAC inhibitors and by over-expression of transcription factors known to increase *BDNF* expression, indicating that transcription of the transgenic reporter is regulated similarly to the endogenous *BDNF* gene.

**Conclusions:**

Generated rBDNF-hRluc-EGFP BAC cell lines respond to known modulators of BDNF expression and could be used for screening of compounds/small molecules or transcription factors altering BDNF expression.

## Background

Brain derived neurotrophic factor (BDNF), a nerve growth factor family member [1], has been shown to have important roles in the development and functioning of nervous system [2]. During development, BDNF supports survival and differentiation of distinct neuronal subpopulations [1,3,4]. In adulthood, BDNF has been shown to have effects in activity-dependent synaptic plasticity including learning and long-term potentiation [5], pain modulation [6], synaptogenesis [7,8] and regulation of metabolism [9].

*BDNF* gene has complex transcriptional regulation with different untranslated 5′ exons spliced to a common protein coding 3′ exon. Nine different promoters (I-IX) controlling transcription from nine or eleven 5′ exons, in rodents or humans respectively, and two different polyadenylation sites give rise to a range of mRNAs [10-12]. *BDNF* transcription has been shown to be regulated by a multitude of transcription factors (reviewed in [13]), for instance promoter I by cAMP response element binding protein (CREB) [14], upstream stimulatory factors (USF) [14], myocyte enhancer factor 2D (MEF2D) [15], nuclear factor kappa beta (NFκB) [16], basic helix-loop-helix (bHLH)-PAS transcription factor neuronal PAS domain protein 4 (NPAS4) and aryl hydrocarbon receptor nuclear translocator 2 (ARNT2) heterodimer (NPAS4-ARNT2) [17,18]; promoter II by repressor element-1 transcription factor (REST) [19,20]; promoter IV by CREB [21,22], calcium response factor (CaRF) [23], USF-s [18,24], methyl CpG binding protein 2 (MeCP2) [25], NFκB [26], bHLHB2 [27], and NPAS4-ARNT2 heterodimer [17,18], MEF2C [28]; promoter IX by CREB and NPAS4-ARNT2 heterodimer [18]. Due to the presence of many promoters and resulting large number of transcripts with the same protein coding sequence but alternating 5′ and 3′ untranslated regions, *BDNF* expression is temporally and spatially controlled in different tissues [11,12,29], developmental stages [30] and within different cell compartments [31-33]. Additionally, the *BDNF* gene locus also encompasses the *antisense BDNF* gene (*BDNFOS*) [12,34,35] with a complex splicing and expression pattern. Transcripts of the *antisense BDNF* gene have been shown to form dsRNA duplexes with *BDNF* transcripts [12] and regulate BDNF levels [35]*in vivo*.

Alterations in *BDNF* expression have been associated with several neurodegenerative disorders. *BDNF* expression has been shown to be decreased in brains of Alzheimer’s [36], Parkinson’s [37,38] and Huntington’s disease [39] patients. Changes of BDNF levels are accompanied by several other pathologies, like neuropsychiatric disorders, obesity, impairment of learning and memory, neuropathic pain and epileptogenesis [2]. Due to BDNF involvement in nervous system disorders, it has been of great interest to use it as a therapeutic [40]. Unfortunately, direct use of recombinant BDNF protein is problematic due to its low serum half-life, poor penetration across blood brain barrier and low diffusion properties in tissues. Delivery of BDNF into the brain using viral vectors can have problems with vector toxicity, expression dosage and insertional mutagenesis. These problems have promoted screening of drug candidates that could promote expression of endogenous BDNF [41]. HDAC inhibitors are one class of drugs that have been shown to mediate their effect on memory and synaptic plasticity in models of nervous system disorders through increase in *BDNF* expression [42,43].

Bacterial artificial chromosomes (BACs) are large capacity vectors which are easy to maintain and modify using homologous recombination in *E. coli*[44]. Due to their large size, BACs can incorporate whole gene genomic loci while at the same time being easier to handle and modify than yeast artificial chromosomes (YACs). BACs have been used to create transgenic mice and cell lines for studying protein function [45], expression regulation [46-48] and for use in high-throughput screening of gene expression modulators [49].

Our group has previously created transgenic mice using BACs containing human [50] or rat [51]*BDNF* genomic sequences. Transgenes in these mice recapitulated endogenous *BDNF* expression patterns in different tissues. In the current study, we have generated transgenic cell lines expressing humanised *Renilla* luciferase (hRluc)-EGFP fusion reporter gene under the control of rat *BDNF* gene regulatory sequences. To this end we used BAC containing rat *BDNF* gene locus with BDNF protein coding region replaced with the hRluc-EGFP coding sequence. These transgenes maintain transgene episomally in high numbers and express reporter gene at high levels. Reporter gene is induced in response to rise in intracellular calcium levels, treatment with different HDAC inhibitors and overexpression of NPAS4-ARNT2 heterodimer or constitutively active CREB1 (VP16-CREB) that are known to regulate *BDNF* expression. These transgenic cell lines could be used for screening drug candidates or transcription factors that modulate *BDNF* expression.

## Results

### Generation of rBDNF-hRluc-EGFP HeLa stable cell lines

HeLa cell line was chosen for generation of rBDNF-hRluc-EGFP cell lines because its relatively carefree growth conditions and fast growth are good properties for transgenic cell line. Endogenous human *BDNF* gene was also expressed in HeLa cell line showing that signaling pathways regulating *BDNF* expression were active in HeLa cells (see below). The hRluc-EGFP fusion reporter was used because EGFP fluorescence was useful for initial screening and subcloning of transgenic cell lines by fluorescence microscopy and FACS. However for screening of substances or transcription factors regulating *BDNF* expression, *Renilla* luciferase luminescence detection is more sensitive and gives less background signal than fluorescence based detection methods [52]. *Renilla* luciferase also has commercial live cell substrates that allow for repeated measurements of the treated cells making it easier to assay the time dependent effect on the reporter expression while conserving reagents.

**Figure 1:**
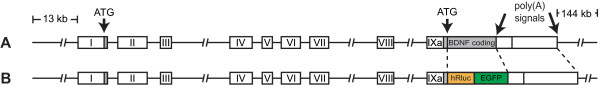
**Schematic drawing of the rBDNF-hRluc-EGFP-BAC construct used for generation of HeLa cell-lines expressing the transgene. (A)** The BAC used contained the rat BDNF locus spanning 13 kb upstream of the first exon and 144 kb downstream of the last polyadenylation signal. The exonic structure of rat BDNF gene is adapted from [11]. **(B)** The coding region of BDNF was replaced by homologous recombination with sequence coding for humanised *Renilla* luciferase, Ala-Ala-Ala-Thr linker and EGFP fusion protein. Protein coding regions are shown as gray (BDNF), orange (hRluc) or green (EGFP) boxes. Untranslated regions are shown as white boxes.

rBDNF-hRluc-EGFP BAC construct used for generating cell lines was created using BAC clone that contains rat *BDNF* locus spanning 13 kb upstream of the first exon and 144 kb downstream of the last polyadenylation signal (Figure 1A). BAC clone was modified by homologous recombination to: (i) replace BDNF coding sequence with sequence coding for humanised *Renilla* luciferase, Ala-Ala-Ala-Thr linker and EGFP fusion protein (Figure 1B) and (ii) replace *CAT* gene in BAC vector by *neo* cassette to confer resistance to G418 for positive selection during cell line generation. The final rBDNF-hRluc-EGFP BAC construct was transfected into HeLa cells by nucleofection and G418 was applied for selection.

Following two months of G418 selection, FACS analysis of polyclonal cell population showed that 15% of cells were positive for EGFP signal. Luciferase signal measured in cell lysate was 10^4^ times over the HeLa background signal (data not shown). By FACS assisted cell sorting a number of single cell clones were established displaying varying levels of transgene expression. Six cell lines were chosen for subsequent analysis: 1A4s2, 1A4s3, 2A4, 2B2s, 3E2s and 3G4s.

### rBDNF-hRluc-EGFP cell lines express hRluc-EGFP reporter gene

**Figure 2:**
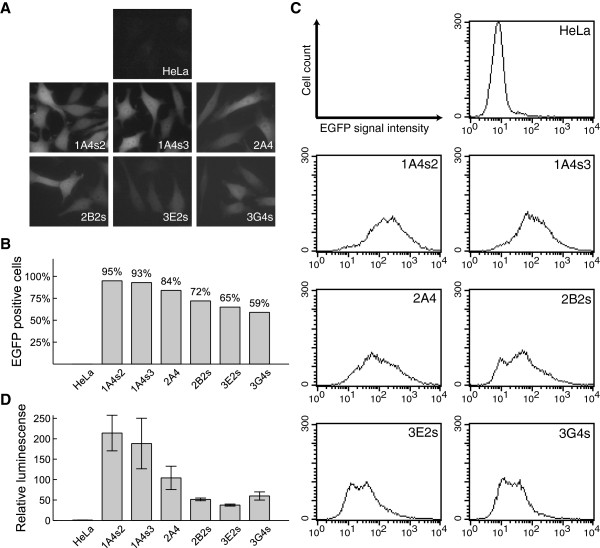
**Detection of hRluc-EGFP reporter expression in rBDNF-hRluc-EGFP cell lines. (A)** Fluorescence microscopy images of rBDNF-hRluc-EGFP cell lines. **(B)** Percentage of EGFP positive cells in different rBDNF-hRluc-EGFP BAC cell lines counted by fluorescence-activated cell sorting (FACS). **(C)** Histograms showing the variability of reporter gene expression level within a cell population of different rBDNF-hRluc-EGFP cell lines counted by FACS. **(D)** Detection of *Renilla* luciferase enzymatic activity in rBDNF-hRluc-EGFP cell lines relative to the background signal measured in parental HeLa cells. Enduren luciferase live cell substrate was added with fresh culture medium to rBDNF-hRluc-EGFP cells and HeLa cells and *Renilla* luciferase luminescence was measured after 12 hour incubation. Results are normalised to cell viability assayed by cellular ATP levels after 12 hour incubation. Error bars indicate SD of three independent experiments.

In all six cell lines, EGFP signal was detected by fluorescence microscopy in live cells, and hRluc-EGFP fusion protein was distributed diffusely all over the cell (Figure 2A). Flow-cytometric analysis of the six cell lines showed that the percentage of EGFP positive cells in population varied from 95 to 59 percent (Figure 2B). Additionally there was a variance over several log units in reporter expression level within a cell line (Figure 2C). This prompted us to analyse the stability of reporter expression in time. For this we passaged the cell lines in media containing increasing concentrations of selective antibiotic G418 (0, 200, 400, 800 and 1200 μg/ml). If no antibiotic was added to the growth medium then the proportion of EGFP positive cells in different cell lines decreased 1.5 to 10 times in three weeks. At the same time the proportion did not change substantially when cells were grown in medium containing 800 or 1200 μg/ml of G418 (data not shown).

Next we measured the activity of *Renilla* luciferase in live cells using Enduren substrate and normalised it to cellular ATP levels. The signals obtained from the six cell lines were 35 to ~200 times over the background signal of the parental HeLa cells (Figure 2D). Based on reporter expression level we divided the cell lines into two groups: the group with high reporter expression includes the cell lines 1A4s2, 1A4s3 and 2A4; the low reporter expression group consists of the cell lines 2B2s, 3E2s and 3G4s. In the first group luciferase signal was ~100-200 times above background and in the second group around ~35-60 times above background.

**Figure 3:**
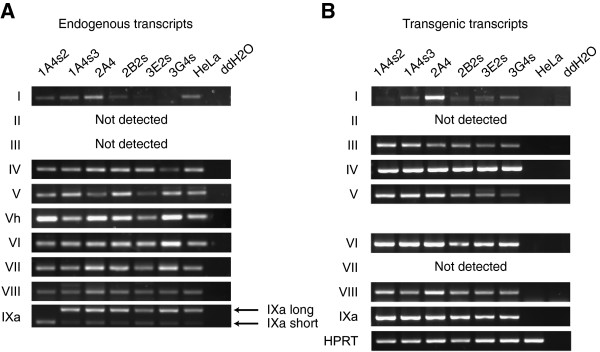
**Expression of alternative 5′ exon-specific mRNAs transcribed from rBDNF-hRluc-EGFP cell lines and HeLa cells.** Expression of alternatively spliced 5′ exon specific transcripts form **(A)** endogenous BDNF and **(B)** rBDNF-hRluc-EGFP BAC reporter construct in different rBDNF-hRluc-EGFP cell lines and parental HeLa cells. Endogenous IXa long and short transcripts correspond to human BDNF transcripts IXabcd and IXabd in [12].

Having detected both, *Renilla* luciferase luminescence and EGFP fluorescence, of the fusion reporter protein we decided to determine which of the alternative chimaeric rat BDNF hRluc-EGFP mRNAs are transcribed in the six cell lines. Expression of endogenous human *BDNF* transcripts in the 6 cell lines were similar to parental HeLa cells with a few exceptions: transcript I was not expressed in 3G4s cell line and transcript IXa long (corresponding to transcript IXabcd in [12]) was not expressed in 1A4s2 cell line (Figure 3A). Using RT-PCR analysis we were able to detect transgenic transcripts I, III, IV, V, VI, VIII and IXa in all cell lines (Figure 3B). Transgenic transcript I was expressed at very low levels in five out of six cell lines, its levels were elevated in 2A4 cells. Neither endogenous nor transgenic transcript II was detected in cell lines or parental HeLa cells. While overall expressions of endogenous and transgenic transcripts were similar there were some differences. First, endogenous transcript III was not expressed in any of the cell lines while transgenic transcript III was expressed in all cell lines. Second, transgenic transcript VII was not expressed in any of the cell lines although being expressed endogenously. Altogether these data show that correctly spliced transgenic rat *BDNF* hRluc-EGFP mRNAs are transcribed and functional hRluc-EGFP fusion protein is expressed in rBDNF-hRluc-EGFP cell lines.

### Transgene is maintained in rBDNF-hRluc-EGFP cell lines as a high copy number episome

**Figure 4:**
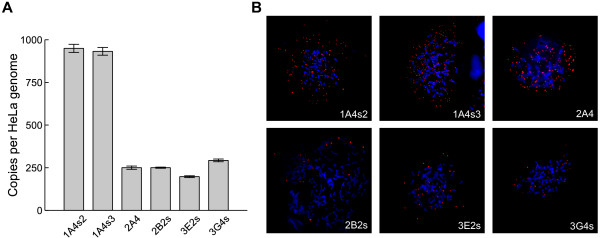
**Analysis of transgene copy number and chromosomal state. (A)** qPCR analysis of transgene copy number in different rBDNF-hRluc-EGFP cell lines. Error bars show SE of three technical replicates. **(B)** FISH analysis of reporter construct chromosomal status in different rBDNF-hRluc-EGFP cell lines. Hybridisation was performed with rBDNF-hRluc-EGFP BAC specific probe (red signal) and DNA was stained with Hoecht 33342 (blue signal).

Since transgene integration site and copy number can influence reporter gene expression from the transgene, we aimed to determine the copy number and chromosomal state (integrated or episomal) of rBDNF-hRluc-EGFP BAC DNA in cell lines. qPCR analysis using copy number standard showed that transgene copy number varied up to five times amongst the different cell lines. Over 900 transgene copies per HeLa genome were present in 1A4s2 and 1A4s3 cell lines and ~190-300 transgene copies in 2A4, 2B2s, 3E2s, 3G4s cell lines (Figure 4A). The status of transgene DNA was analysed by fluorescent *in situ* hybridisation (FISH) with rBDNF-hRluc-EGFP BAC specific probe. As demonstrated in Figure 4B, transgenic BAC DNA was maintained episomally in all cell lines – the rBDNF-hRluc-EGFP BAC specific hybridisation signals were localised near chromosomes, but integration was not detected. Transgene copy numbers per cell also varied highly between cells of the same cell line. In conclusion, the obtained rBDNF-hRluc-EGFP cell lines contain relatively high numbers of transgenic BAC DNA per cell that replicates episomally.

### Elevated intracellular calcium induces reporter gene expression in rBDNF-hRluc-EGFP cell lines

**Figure 5:**
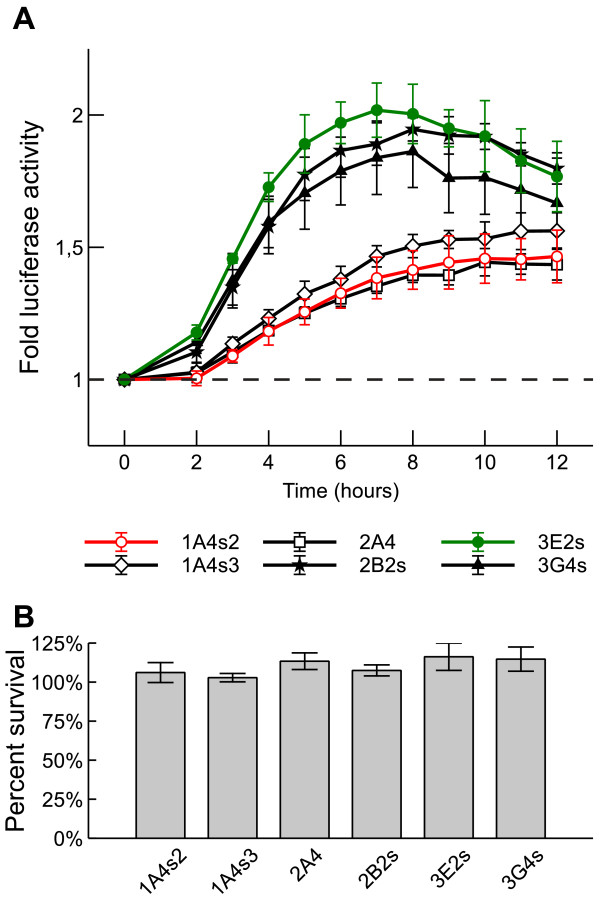
**Treatment with ionomycin induces reporter expression in different rBDNF-hRluc-EGFP cell lines. (A)** Luciferase activity in 1 μM ionomycin treated cells relative to vehicle (0.1% DMSO) treated cells (dashed line) at each time point, measured in live cells during 12 hours. *Renilla* luciferase luminescence was measured using Enduren substrate 2–12 hours after the start of the treatment at one hour intervals. Error bars indicate SD of three independent experiments. **(B)** Cell viability in ionomycin treated cells relative to vehicle treated cells after 12 hours in different cell lines. Viability was assayed by cellular ATP levels. Error bars indicate SD of three independent experiments.

*BDNF* promoters contain several Ca^2+^ responsive regulatory elements [14,18,21-24,53] and BDNF levels *in vivo* are induced by neural activity related Ca^2+^ influx into neurons [54]. To test if transgene is induced by elevated intracellular Ca^2+^ levels in different cell lines we treated cells for 12 hours with 1 μM ionomycin, a calcium ionophore known to induce *BDNF* expression in neurons [55], and monitored *Renilla* luciferase signal using Enduren live cell luciferase substrate during this period. Ionomycin treatment induced reporter gene expression in all cell lines compared to vehicle treated control. As shown in Figure 5A, the relative increase of luminescence signal was higher in cell lines with low reporter expression: 3E2s, 2B2s and 3G4s. In these three cell lines the fold change reached its peak after eight hours when it was 2.00 (p < 0.01); 1.95 (p < 0.01); and 1.86 (p < 0.05) respectively. In the cell lines with high reporter expression the fold change remained smaller and the maximum values for cell lines 1A4s2, 1A4s3 and 2A4 were 1.46 (p < 0.05); 1.56 (p < 0.01) and 1.43 (p < 0.01) respectively. To exclude the possibility that ionomycin affects cell viability we measured the cell viability levels of control and ionomycin treated cells after 12 hours. No significant decrease in cell viability was detected in response to ionomycin treatment (Figure 5B). These results demonstrate that reporter expression in rBDNF-hRluc-EGFP cell lines is regulated by changes in intracellular calcium level.

### HDAC inhibitors induced reporter gene expression in rBDNF-hRluc-EGFP cell lines

**Figure 6:**
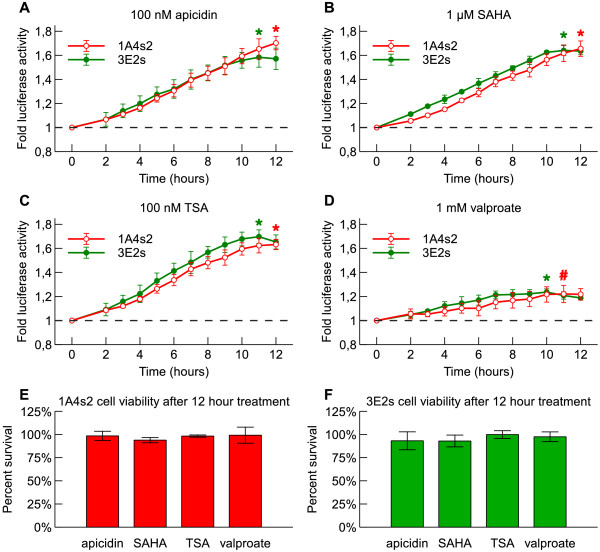
**Treatment with HDAC inhibitors induces reporter expression in 1A4s2 and 3E2s rBDNF-hRluc-EGFP cell lines. (A, B, C, D)** Fold luciferase activity in HDAC inhibitor treated cells relative to vehicle treated cells (dashed line) measured in live cells during 12 hours. 1A4s2 and 3E2s cells were treated with HDAC inhibitors apicidin (100 nM) **(A)**, SAHA (1 μM) **(B)**, TSA (100 nM) **(C)**, sodium valproate (1 mM) **(D)** and vehicle control (0.1% DMSO or water) together with Enduren live cell substrate. Error bars indicate SD of three independent experiments (* p < 0.01, # p < 0.05, relative to vehicle treated control). **(E, F)** Cell viability in HDAC inhibitor treated cells relative to vehicle treated cells after 12 hours of treatment in 1A4s2 cell line **(E)** and 3E2s cell line **(F)**. Viability was assayed by cellular ATP levels. Error bars indicate SD of three independent experiments.

Due to interest in finding low molecular weight substances regulating *BDNF* expression we sought to establish whether our cell lines could be used for screening of substances modulating *BDNF* expression. HDAC inhibitors are a class of drugs that inhibit histone deacetylases – group of enzymes that deacetylate histones and non-histone proteins. It has been shown that certain HDAC inhibitors have antidepressant actions and regulate *BDNF* expression [56], for example valproate [42,57-59], TSA [58,60] and SAHA [61]. Since it would be of interest to use our cell lines for screening of other compounds that could epigenetically regulate *BDNF* expression, we tested response of the reporter gene in BAC cell lines to four HDAC inhibitors. Two cell lines were chosen for treatments, higher copy number cell line 1A4s2 and lower copy number cell line 3E2s. The cells were treated with 100 nM apicidin, 1 μM SAHA, 100 nM TSA and 1 mM sodium valproate for 12 hours while assaying reporter gene expression during that time using Enduren live cell *Renilla* luciferase substrate.HDAC inhibitors increased reporter gene expression in both cell lines compared to vehicle treated control. 100 nM apicidin increased reporter gene expression in 1A4s2 and 3E2s cell lines 1.70 and 1.58 fold at 12 hours and 11 hours of treatment, respectively (both p < 0.01, Figure 6A). 1 μM SAHA treatment increased reporter gene expression in 1A4s2 and 3E2s cell lines 1.65 and 1.64 fold at 12 hours and 11 hours, respectively (both p < 0.01 Figure 6B). 100 nM TSA treatment increased reporter gene expression in 1A4s2 and 3E2s cell lines 1.63 and 1.70 fold at 12 hours and 11 hours, respectively (both p < 0.01, Figure 6C). 1 mM sodium valproate increased reporter gene expression in 1A4s2 and 3E2s cell lines 1.22 and 1.24 fold at 11 hours and 10 hours, respectively (p < 0.05 and p < 0.01, Figure 6D). None of the used HDAC inhibitors showed significant effect on cellular survival after 12 hours of treatment in either cell line (Figure 6E and F). Taken together, these results show that HDAC inhibitors upregulate reporter gene expression in rBDNF-hRluc-EGFP cell lines 1A4s2 and 3E2s.

### VP16-CREB and NPAS4-ARNT2 transcription factors increased transgene expression in BAC transgenic cell lines

**Figure 7:**
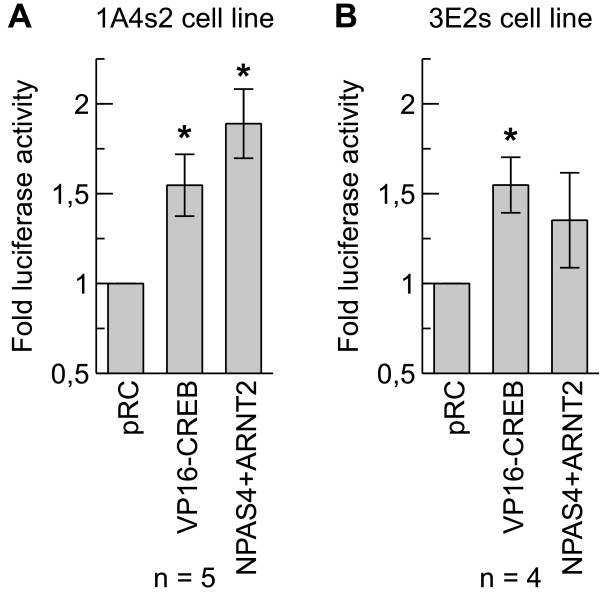
**Overexpression of VP16-CREB and NPAS4 + ARNT2 induces reporter expression in 1A4s2 and 3E2s rBDNF-hRluc-EGFP cell lines. (A, B)** 1A4s2 **(A)** and 3E2s **(B)** cells were transfected with constructs expressing VP16-CREB, NPAS4 and ARNT2 (at 1:1 ratio) transcription factors or empty vector control pRC. *Renilla* luciferase luminescence was assayed 24 hours after transfection and fold luciferase activity relative to empty vector control (pRC) was calculated. Error bars indicate SD. (n – number of independent experiments, * p < 0.01, relative to empty vector control).

*BDNF* is regulated by 9 different promoters containing binding sites for different transcription factors regulating expression of different *BDNF* transcripts. Of these transcription factors CREB and bHLH transcription factor heterodimer NPAS4-ARNT2 have been shown to regulate *BDNF* expression from various promoters [18,21,22]. To test if the transgenic cell lines could be used for screening of transcription factors that induce *BDNF* transcription we transfected cell lines 1A4s2 and 3E2s with constructs expressing VP16-CREB (constitutively active form of CREB1, fused with viral transactivation domain) [62] or NPAS4 and ARNT2, or with empty pRC vector for comparison.24 hours after transfection VP16-CREB transcription factor increased transgene expression in 1A4s2 and 3E2s cell lines 1.55 (p < 0.01) fold. Transcription factor NPAS4 together with ARNT2 increased reporter expression in 1A4s2 and 3E2s cell lines 1.89 (p < 0.01) and 1.35 fold, respectively (Figure 7A and B). These results show that VP16-CREB and NPAS4-ARNT2 heterodimer increase transgene expression in 1A4s2 and 3E2s rBDNF-hRluc-EGFP cell lines.

## Discussion

In the current study, we have developed rBDNF-hRluc-EGFP reporter cell lines in HeLa background, using bacterial artificial chromosome (BAC) containing rat *BDNF* genomic locus with protein coding region replaced with hRluc-EGFP fusion reporter gene, for studying the regulation of the *BDNF* gene and for analysis of the effect of different compounds and transcription factors on *BDNF* expression. Generation of transgenic mice for studying *BDNF* regulation using large transgenic constructs by us [50,51] and others [63] has shown that use of BAC (or YAC) transgenic constructs helps to better recapitulate endogenous *BDNF* expression. It has also been demonstrated that using BAC constructs for generating cell lines helps to avoid transgene integration specific effects and provides levels and timing of transgene expression that mimic that of the endogenous gene [64]. The large rat *BDNF* genomic locus contained in the BAC construct used in this work should include regulatory elements positioned further away from *BDNF* gene and help to better emulate endogenous *BDNF* expression.

Out results show that rBDNF-hRluc-EGFP reporter construct was maintained extrachromosomally in high copy numbers in all established cell lines. Previously it has been shown that plasmids containing matrix attachment regions (MARs) are maintained in double minute extrachromosomal elements in HeLa cells [65]. These are autonomously replicating extrachromosomal elements that are up to a few Mb in size [66], have been known to be associated with active histones [67] and can be purified by histone immunoprecipitation [68]. MARs and scaffold attachment regions (SARs), together named as S/MARs, are regions on the DNA which attach to nuclear matrix and have been associated with functions such as anchoring of DNA, maintenance of nuclear architecture, regulation of transcription and replication. It has been estimated that S/MARs are spaced on average 70 kb from each other in mammalian genomes [69]. Given the large size of *BDNF* locus contained in BAC construct used in this study (207 kb) it is probable that it contains S/MAR elements enabling extrachromosomal maintenance in HeLa cells. We used SMARTest tool [70] to predict the existence of 6 candidate sites in the genomic locus included in rBDNF-hRluc-EGFP BAC which exhibit S/MAR like characteristics (data not shown). It would be of interest to study which regions of the *BDNF* BAC used in this study are responsible for its extrachromosomal maintenance.

We detected expression of almost all 5′ exon-specific BDNF mRNAs in the rBDNF-hRluc-EGFP cell lines showing that the entire *BDNF* gene is maintained in inserted transgene. We also observed stable and copy number dependent expression of hRluc-EGFP fusion reporter protein by fluorescence and luminescence based methods. Previously it has been shown that protein expression in BAC transgenic cell lines is proportional to transgene copy numbers [71]. BAC-derived *BDNF* gene is not highly expressed, as shown by transgenic animals previously produced in our lab where one or two copies of transgenic *BDNF* BAC construct were inserted in the genome. In contrast, the high copy numbers of transgene in the cell lines developed in this study directed high levels of reporter gene expression.

We used hRluc-EGFP fusion protein, analogous to the Rluc-GFP reporter used previously by [72], as a reporter in the BAC construct. The use of hRluc-EGFP protein makes it possible to apply both fluorescence and more sensitive luminescence based methods for reporter detection. While EGFP fluorescence could be used for measuring reporter expression via flow cytometry, use of live cell substrates allow for sensitive detection of *Renilla* luciferase signal which could be advantageous for high-throughput screening procedures. The half-life of *Renilla* luciferase is 3–4 hours and the half-life of EGFP is about 26 hours [73] in mammalian cells. However, stability of the hRluc-EGFP fusion protein is not known. It is probable that the induction of measurable reporter activity in response to different treatments underestimates the true effect on transcriptional activity due to slow turnover of the hRluc-EGFP fusion protein, suggesting that it would be important to develop a less stable hRluc-EGFP fusion protein in future studies.

To assess the suitability of the cell lines generated by us for use in studying *BDNF* gene regulation, the cells were treated with stimuli known to induce *BDNF* expression. Ionomycin induces *BDNF* expression through Ca^2+^ mediated signalling pathways [54], HDAC inhibitors through increasing histone acetylation leading to transcriptionally active chromatin around subset of genes, including *BDNF*[56,74]. Transcription factor CREB and NPAS4-ARNT2 heterodimer have been shown to promote transcription from several *BDNF* promoters [18,21,22]. As expected, treatment with different modulators of *BDNF* expression induced expression of hRluc-EGFP fusion reporter protein. The effect of treatment with ionomycin or HDAC inhibitors was different when comparing high and low transgene copy number cell lines. Ionomycin induced reporter expression to a higher extent in lower copy number cell lines than in higher copy number cell lines. However, treatment of high and low transgene copy number cell lines with different HDAC inhibitors induced transgene expression similarly in both cell lines regardless of the used inhibitor. The apparent copy number dependent effect on reporter induction by ionomycin might be explained by higher number of transcription factor response elements in high copy number cell lines competing for limited supply of Ca^2+^ dependent transcription factors. In contrast, the effect of HDAC inhibitors on gene expression is more general, regulating expression of large number of genes, and they may act by inducing transgene expression independent of copy number.

Since BDNF has been primarily studied as a neuronal gene, the non-neuronal nature of the generated transgenic cell lines sets certain limitations to their use in studying neuron-specific regulation of BDNF expression. For example, BDNF mRNAs have been known to be transported to dendrites [75,76] and their translation there to be regulated in response to local synaptic signalling [77], regulatory steps that are not recapitulated in our cell lines. Also, neuronal stimuli known to regulate BDNF expression, for example depolarisation by potassium [54] or glutamate [78], do not recapitulate in HeLa background. Therefore, modulators that have been found to regulate transgene expression in these transgenic cell lines should be also verified in neuronal background. However, the active transcription of endogenous BDNF mRNAs in HeLa cells and the robust nature of the HeLa cells make these cell lines convenient tools for screening of factors regulating BDNF expression.

## Conclusions

In conclusion, we have generated a rBDNF-hRluc-EGFP BAC cellular reporter model for use in studying *BDNF* regulation. Transgene is maintained in cell lines extrachromosomally as high copy number episome. High transgene copy number makes it possible to reliably detect reporter expression. Transgene expression is induced in response to known modulators of *BDNF* expression making these cell lines useful for further studies of *BDNF* regulation.

## Methods

### Constructs

BAC clone CH230-106 M15 containing rat BDNF gene was purchased from Chori BACPAC Resources. Clone CH230-106 M15 contains rat genomic DNA region spanning the *BDNF* gene locus cloned into the EcoRI site of pTARBAC2.1 vector. The vector carried chloramphenicol resistance and resided in *E. coli* host strain DH10B (recA^−^, recBC^+^). Sequence of the BAC clone CH230-106 M15 was obtained from NCBI GeneBank [GenBank:AC108236]. Vectors pCDNA3.1-NPAS4, pCDNA3.1-ARNT2 and pACT-CREB1 (containing VP16 viral transcription activation domain) have been described previously [18].

### Homologous recombination

BAC modifications using Red/ET homologous recombination were performed according to the BAC Modification Counter-Selection System protocol (Gene Bridges GmbH). For amplification of inserts, 75-mer oligonucleotides were synthesised (Proligo). The 5′-end of each oligonucleotide contained 50 nucleotides of homology region shared by the target BAC and a linear insert followed by a 25 nucleotide primer for PCR amplification of the linear insert from the template. Where necessary, linker sequence was added between homology arm and primer sequences. Inserts for homologous recombination were amplified by PCR using Expand Long Template PCR system (Roche) or Hot GyroPol PCR system (Solis BioDyne). The synthetic humanised version of *Renilla* luciferase reporter gene (hRluc), the red-shifted variant of wild-type *Aequorea* green fluorescent protein (EGFP) reporter gene and SV40-Neo^r^-polyA cassette were amplified from pTK-hRluc (Promega), pEGFP-N1 (Clontech) and pEGFP-C1 vectors (Clontech), respectively. Following PCR primers were used for insert synthesis – hRLuc: sense 5′-CCT GTT CTG TGT CTG TCT CTG CTC CTT CCC ACA GTT CCA CCA GGT GAG AAG AGT GAT GGC TTC CAA GGT GTA CGA CCC CG-3′, antisense 5′-ATA CAA ATA GAT AAT TTT TGT CTC AAT ATA ATC TAT ACA ACA TAA ATC CAT TAC TGC TCG TTC TTC AGC ACG CGC T-3′; EGFP: sense 5′-TGG GTA AGT ACA TCA AGA GCT TCG TGG AGC GCG TGC TGA AGA ACG AGC AGG CCG CCG CCG CCA CCA TGG TGA GCA AGG GCG AGG AGC TG-3′, antisense 5′-ATA CAA ATA GAT AAT TTT TGT CTC AAT ATA ATC TAT ACA ACA TAA ATC CAT TAC TTG TAC AGC TCG TCC ATG CCG A-3′; SV40- Neo^r^ -polyA cassette: sense 5′-CAC CAT AAT GAA ATA AGA TCA CTA CCG GGC GTA TTT TTT GAG TTA TCG AGA TTT TCA GGA GCT AAG GAA GCT AAA TTC AAA TAT GTA TCC GCT CAT GAG A-3′, antisense 5′-ATT CAT CCG CTT ATT ATC ACT TAT TCA GGC GTA GCA ACC AGG CGT TTA AGG GCA CCA ATA ACT GCC TTT TTT ATT CTG TCT TTT TAT TGC CGT C-3′. BAC was modified by first replacing BDNF protein coding region with hRluc coding sequence, then inserting Ala-Ala-Ala-Thr linker and EGFP coding sequences to the end of hRluc sequence and finally by replacing the *CAT* gene in BAC vector with SV40-Neo^r^-polyA cassette. The modified BAC containing colonies were screened by colony PCR and colony hybridisation and further verified by restriction analysis and sequencing.

### Cell culture and transfection

HeLa (DSMZ) human cervical cancer cells were propagated in DMEM (Dulbecco’s Modified Eagle Medium; PAA) supplemented with 10% fetal bovine serum (PAA), penicillin (PAA) and streptomycin (PAA) at 37°C in 5% CO2. For BAC DNA transfection 1 μg of BAC DNA purified with Large-Construct Kit (Qiagen) was mixed with 5 × 10^6^ HeLa cells and transfection was performed using Amaxa Nucleofector program A-28 and Cell Line Nucleofector Kit R (Amaxa). 72 hours later 400 μg/ml G418 (Sigma) was added to the growth medium to select for BAC containing cells. After two months of selection, single EGFP-positive clones were isolated with FACSAria cell sorting system (Becton-Dickinson). The cell lines were routinely grown in medium containing 800 μg/ml G418. For transfection of plasmid constructs cells were seeded into white 96-well clear bottom microtiter plate (Greiner Bio-One) at 10 000 cells per well in a volume of 200 μl of G418-containing culture medium. The next day medium was replaced with 100 μl of medium without G418 and cells were transfected with GenJet Hela transfection reagent (SignaGen Laboratories) using 100 ng of DNA per well at 1:3 DNA to lipid ratio. NPAS4 and ARNT2 constructs were cotransfected at 1:1 ratio. Five hours post-transfection medium was replaced with 200 μl of G418-containing medium.

### Drug treatments

One day before treatments, cells were seeded into white 96-well clear bottom microtiter plates (Greiner Bio-One) at 10 000 cells per well. The next day medium was replaced with 75 μl of fresh medium containing ionomycin, apicidin, sodium valproate (all Sigma Aldrich), SAHA or TSA (both Cayman Chemical). All drugs except sodium valproate were dissolved in DMSO and added to cells at a final DMSO concentration of 0.1%. Sodium valproate was dissolved in MilliQ grade water. Appropriate vehicle controls (DMSO or water) were included in all experiments.

### Reporter assays

EGFP was detected by fluorescence microscopy (Axiovert 200 M, Zeiss) and flow cytometry (FACSCalibur, Becton-Dickinson). In flow-cytometric analysis no compensation was used and markers for positive EGFP-signals were set on FL1 vs FL2 dot blot using the autofluorescence diagonal of parental HeLa cells. EGFP-positive cells were identified by divergence from the autofluorescence diagonal towards higher FL1 fluorescence. For monitoring hRluc enzymatic activity in live cells, 30 μM Enduren substrate (Promega) was added to cells at the beginning of drug treatment and luminescence was measured once per hour at 2–12 hour time points. For measuring endpoint hRluc activity, cells were lysed in 20 μl Passive Lysis Buffer (Promega) 24 hours after transfection and subjected to Renilla-Glo Luciferase Assay System (Promega) according to the manufacturer’s instructions. Relative luminescence was measured with GENios Pro plate reader (TECAN). For normalisation and monitoring cell viability ViaLight Plus Cell Proliferation And Cytotoxicity BioAssay Kit (Lonza, USA) were used. For drug treatments, three independent experiments were performed, each in triplicate. Luciferase signal in response to drug treatments was normalised to vehicle control for each time point, means and standard deviations were calculated and t-tests for analysis of statistical significance for indicated time points were performed. For transcription factor transfections, four or five independent experiments were performed, each in triplicate. Luciferase signal in response to expression of transcription factor(s) was normalised to signal in pRC empty vector transfected cells, means and standard deviations were calculated and t-tests for analysis of statistical significance were performed.

### Fluorescence in situ hybridisation

Mitotic blocking was performed by treating cells with 50 ng/ml colcemid (Sigma) for 4 hours. The cells were harvested by shakeoff and subjected to hypotonic treatment with 0.075 M KCl for 15 min. The cells were fixed in methanol:acetic acid (3:1) and used for chromosome slide preparation. Slides were chemically aged and denatured as described [79]. Prior to denaturation and hybridisation, chromosome preparations were treated with RNase A (100 μg/ml in 2 × SSC), pepsin (50 μg/ml in 0.01 N HCl) and 1% formaldehyde (in PBS containing 50 mM MgCl_2_). rBDNF-hRluc-EGFP BAC specific probe was labeled with digoxigenin-11-dUTP (Roche) by nick translation. For each hybridisation 45 ng of the labeled probe was used together with 25 μg of salmon sperm DNA. Hybridisation was carried out by incubating slides in 50% deionised formamide, 2 × SSC, 0.1 M phosphate buffer, 10% dextran sulfate overnight at 37°C in humid chamber. Hybridised probe was detected by affinity reaction with mouse anti-digoxygenin primary antibody (Roche) followed by Alexa 546 conjugated anti-mouse secondary antibody (Life Technologies, USA) and chromosomes were counterstained with Hoecht 33342. Slides were mounted in ProLong Gold anti-fade reagent (Life Technologies, USA) and imaged with Zeiss LSM DUO microscope.

### RNA extraction and RT-PCR

Total RNA from cells was purified with RNeasy Micro kit (Quiagen) as recommended by the manufacturer and treated with DNase I using DNA-free kit (Ambion). First-strand cDNA was synthesised from 5 μg of total RNA with Superscript III reverse transcriptase (Life Sciences) according to manufacturer’s recommendations. PCR reactions were performed with HotFire polymerase (Solis Biodyne) in a volume of 10 μl containing 1/80 of reverse transcription reaction as a template. Human *BDNF* 5′ exons’ specific primers have been described previously [12]. Rat *BDNF* 5′ exons’ specific primers have been described previously [11] and were used in combination with *hRluc* specific antisense primer 5′-GTA CTT GTA GTG ATC CAG GAG GCG AT-3′.

### Genomic DNA extraction and quantitative PCR

Genomic DNA was extracted from cells by proteinase K digestion and phenol:chloroform extraction followed by ethanol precipitation and resuspension overnight in TE (pH 8.0). Genomic DNA concentration was quantified with UV spectrophotometer (NanoDrop) and diluted to 16 ng/μl for qPCR. For standard curve, a series of mixtures in which the number of pEGFP-C1 (Promega) plasmid molecules ranged from 128 to 1024 copies per HeLa genome were prepared using HeLa genomic DNA. 32 ng of genomic DNA from different cell lines or copy number standards were subjected to quantitative PCR. Quantitative PCR was performed on Roche LightCycler 2.0 using qPCR Core kit for SYBR® Green I No ROX (Eurogentec). qPCR reactions with copy number standards were performed in duplicate. qPCR reactions with cell line genomic DNAs were performed in triplicate. Melting curve analysis was carried out at the end of cycling to confirm amplification of a single PCR product. Following *EGFP* and human *TRKB* (genomic control) specific PCR primer sets were used: *EGFP* sense 5′- CAG AAG AAC GGC ATC AAG GTG-3′, antisense 5′- TGG GTG CTC AGG TAG TGG TTG -3′; *TRKB* sense 5′- CAC AGG GCT CCT TAA GGA TAA C -3′, antisense 5′- GCA CAG TGA GGT TGA CAG AAT C-3′. Copy number estimates were calculated with qBASEPlus 2.6 software (Biogazelle) using *EGFP* as target and *TRKB* as reference.

## Abbreviations

BAC: Bacterial artificial chromosome; BDNF: Brain derived neurotrophic factor; FISH: Fluorescence in situ hybridisation; HDAC: Histone deacetylase; *hRluc*: Humanised *Renilla* luciferase; MAR: Matrix attachment region; SAR: Scaffold attachment region; YAC: Yeast artificial chromosome.

## Competing interests

The authors declare that they have no competing interests.

## Authors’ contributions

KJ performed reporter expression analysis, qPCR analysis of transgene copy number, fluorescent in situ hybridisation, drug treatments, transcription factor transfection, data analysis of the results and drafting of the manuscript. MS carried out BAC transfection, established cell lines, performed FACS sorting and analysis, fluorescence microscopy, ionomycin treatments and contributed to the initial design of the study. TAP prepared the rBDNF-hRluc-EGFP BAC construct and contributed to the initial design of the study. TT conceived and coordinated the study. All authors contributed to the preparation of the manuscript. All authors read and approved the final manuscript.
